# Antioxidant Potential of Probiotics and Postbiotics Derived from Lactic Acid Bacteria and Their Impact on Foods

**DOI:** 10.3390/foods15071253

**Published:** 2026-04-06

**Authors:** Radoslav Abrashev, Ekaterina Krumova, Nikoleta Atanasova, Lili Dobreva, Maria Angelova, Svetla Danova

**Affiliations:** 1 Department of Mycology, Stephan Angeloff Institute of Microbiology, Bulgarian Academy of Sciences, Academician G. Bonchev 26, 1113 Sofia, Bulgaria; rabrashev@microbio.bas.bg (R.A.); e_krumova@microbio.bas.bg (E.K.); mariange@microbio.bas.bg (M.A.); 2 Department of General Microbiology, Stephan Angeloff Institute of Microbiology, Bulgarian Academy of Sciences, Academician G. Bonchev 26, 1113 Sofia, Bulgaria; nikoletaatanasova21@gmail.com (N.A.); lili.ivailova1@gmail.com (L.D.)

**Keywords:** lactic acid bacteria, probiotics, postbiotics, reactive oxygen species, oxidative stress, antioxidant potential, food impact

## Abstract

The diverse health benefits of lactic acid bacteria (LAB) have made them a focal point of research in the fields of food and health sciences. Furthermore, probiotics and postbiotics have been demonstrated to directly or indirectly influence food quality and human health. A substantial body of research has been dedicated to the antimicrobial activity of pro- and postbiotics; however, their antioxidant properties remain relatively unexplored. Although LAB are facultative anaerobes, there are several species that have the potential to undergo aerobic respiration, thereby being exposed to the action of reactive oxygen species (ROS). The resultant oxidative stress has been shown to damage all intracellular molecules, thus requiring the presence of antioxidants in order to counteract this effect. The present review discusses the peculiarities of respiration, the role of ROS, the antioxidant potential of LAB, and the mechanisms underlying their activity. Furthermore, the study explores the antioxidant capacity of probiotics and postbiotics, as well as their role in controlling oxidative stress. The objective of the present review is to provide an overview of the current research on the oxidative stress tolerance and antioxidant capacity of LAB and its impact on food.

## 1. Introduction

Lactic acid bacteria (LAB) are a very important group of microorganisms that play a significant role in various fields, including the food industry, agriculture, and the clinical sector [1]. These bacteria produce lactic acid as a primary metabolic by-product. They are used as starter cultures in the production of many fermented dairy products. Studies have demonstrated that LAB possess significant therapeutic potential, making them indispensable to human health. They exhibit various nutritional, adhesive, and adaptive properties, enabling them to utilize dairy products, fermented foods, fruits, and vegetables as nutrient sources. Furthermore, LAB are known to proliferate extensively within the intestinal tracts of both animals and humans [2].

Many LAB species exhibit probiotic properties. Probiotics (*pro–for* and *bio–life*) are defined as live microorganisms that confer health benefits to the host. They boost the immune system and prevent disease through direct interaction with other microorganisms. They also help to detoxify the intestinal tract and inactivate toxins [3,4]. In recent years, alongside probiotics, prebiotics and symbiotics have become increasingly prevalent. The concept of postbiotics has emerged as a result of the limitations of probiotics’ useful effect in cases of intestinal dysbiosis, immunocompromised people, and reduced populations of beneficial bacteria. According to the International Scientific Association for Prebiotics and Probiotics (ISAPP), postbiotics are defined as ‘preparations of inanimate microorganisms and/or their components that confer a health benefit on the host’ [5,6]. Recent research has indicated that postbiotics can positively impact respiratory diseases and mental health and reduce psychological stress [5,7].

LAB-based probiotics and postbiotics have also been found to exhibit antioxidative effects [1,8]. These natural antioxidants can strengthen the human body’s defense mechanisms against oxidative stress (OS) [9]. The term OS refers to an imbalance between the generation of reactive oxygen species (ROS) in cells and their ability to neutralize them in order to avoid serious damage [10]. Superoxide anions (^•^O_2_^−^) and hydroxyl radicals (^•^OH) are examples of ROS, along with nonradical substances such as singlet oxygen (^1^O_2_) and hydrogen peroxide (H_2_O_2_) and other related species. Various stress factors can accelerate the production of these radicals, overwhelming the antioxidant defenses of cells and tissues. In this context, antioxidant peptides produced by LAB can be highly beneficial for human health.

While considerable interest exists within the scientific community and the public regarding the antioxidant activity of LAB-derived probiotics, several aspects remain underexplored. Specifically, a comprehensive understanding is lacking concerning the antioxidant activity of postbiotics, the mechanisms underlying their protective effects, and their effective application in probiotic foods and supplements. This review overviews recent advances in the study of antioxidant behavior in LAB, focusing on probiotics and postbiotics. After describing the oxygen respiration process in LAB, we emphasize ROS generation and the cellular mechanisms responding to OS. We then discuss the antioxidant defense systems in LAB, specifically referencing enzymatic and non-enzymatic antioxidants. Furthermore, we review recent findings on the antioxidant potential of probiotics and postbiotics and their impact on food quality. The final section outlines future scientific challenges that must be addressed to fully understand cellular responses to oxidative stress and the link between LAB applications and associated health benefits.

## 2. Lactic Acid Bacteria as Probiotics and Postbiotics

### 2.1. LAB as Probiotics

The LAB group includes over 400 species of Gram-positive, non-spore-forming anaerobe to non-respiring, but aerotolerant bacteria, inhabiting natural habitats, as well as found in plants and animals [11]. This physiological group consists of 12–15 genera, including *Streptococcus*, *Lactococcus*, *Pediococcus*, and *Enterococcus*. The biggest genus, *Lactobacillus*, was reclassified into 25 new genera belonging to the family *Lactobacillaceae*. A key common characteristic of LAB is their ability to convert carbohydrates into various lactic acid isomers through fermentation. They are therefore part of the permanent microbiota of fermented foods and the human intestinal tract.

The term “probiotic” has gained significant traction in recent decades and is associated with beneficial effects for humans and animals [1,12]. Probiotic LABs are nonpathogenic microorganisms isolated from various environments, such as foods, animals, plants, and humans [13]. They are commonly considered to be Generally Regarded as Safe (GRAS)/Qualified Presumption of Safety (QPS) and possess distinct characteristics that render them suitable for various applications. Over the years, a variety of probiotic cultures have been administered in the form of fermented dairy products, vegetables, and cereals for the treatment of gastroenteritis and conditions affecting the oral cavity, stomach, small intestine, and large intestine [14].

A significant number of LAB species are used as probiotics to enhance host health. They maintain homeostasis, especially in the gastrointestinal tract (GIT), and positively influence competition with opportunistic bacteria, thereby enhancing digestion, modifying immunity, demonstrating neuromodulation, and producing vitamins and other beneficial substances [4]. Alongside LAB, *Bifidobacteria* and yeasts have also been successfully used as probiotics. Currently, a number of other types of beneficial microbes are being investigated for their probiotic properties [14]. Probiotic bacteria facilitate the reduction in oxidative stress by exhibiting antioxidant characteristics [15]. The antioxidant capacity of probiotic bacteria, particularly lactobacilli, along with their other health-enhancing properties, has been thoroughly studied in recent years.

According to FAO/WHO recommendations, the human gastrointestinal tract is a traditional source of probiotic strains for human consumption. Notably, *Ligilactobacillus salivarius salivarius* subsp. *salicinius* and *Lactobacillus acidophilus* have been isolated from the human intestine, while *Lb. acidophilus*, *Lb. fermentum*, *Lb. gasseri*, *Lb. vaginalis*, *Lb. reuteri*, and *Lb. salivarius* have been identified in human feces and the human stomach, respectively [16]. Probiotic LAB belonging to the genera *Lactococcus*, *Bifidobacterium*, *Leuconostoc*, *Lactobacillales*, and *Pediococcus* have been isolated from fecal samples [17]. These authors highlighted the diet-dependent formation of gut microbiota. The human GIT is a significant reservoir of probiotic bacteria, such as *Lb. rhamnosus* IMC 501, *Lb. paracasei* IMC 502 [18], *Lb. helveticus* BGRA43 [19], *Lb. casei/paracasei* CTC1677, *Lb. casei/paracasei* CTC1678, *Lb. rhamnosus* CTC1679 [20], and *Lactobacillus fermentum* E-3 and E-18 [21]. Zielińska and Kolohyn-Krajewska [16] reported the isolation of probiotics from breast milk and animal GIT, predominantly identified as *Lb. gasseri* and *Lb. reuteri*.

Furthermore, a variety of beneficial microbes have been found in fermented foods, including milk and dairy products, raw fermented meat products, pickled vegetables, and cereal products [2]. Among these, the following have been recognized as effective probiotics: *Lb. rhamnosus* and *Lb. helveticus* (from traditionally fermented Xinjiang cheese) [22], *Lb. plantarum* FC225 (from fermented cabbages) [23], *Lb. plantarum* O7S1 (from fermented olive) [24], *Lb. plantarum* C88 and *Lb. plantarum* MWFLp-182 (from feces) [25,26], and *Lb. plantarum* NJAU-01 (from traditionally dry-cured meat product Jinhua ham) [27].

### 2.2. LAB as Postbiotics

The designation “Postbiotic” can be viewed as a comprehensive term that includes all synonyms and related expressions pertaining to these microbial fermentation components [6]. Postbiotics are derived from non-living microorganisms and/or their constituents, which provide a health advantage to the host. A wide range of ingredients can be classified as postbiotics, such as metabolites including short-chain fatty acids, microbial cell fractions, functional proteins, extracellular polysaccharides, cell lysates, teichoic acid, muropeptides, and peptidoglycan-derived structures [15]. To classify a microbial composition as a postbiotic, both the microorganisms and the content must be identified. Salminen et al. [5] proposed the following criteria.

- An elaborate description of the inactivation procedure along with the matrix;
- Confirmation that the inactivation process has been completed;
- Evidence indicating a health benefit for the host from a controlled, high-quality trial;
- A comprehensive description of the ingredients in the postbiotic preparation;
- An evaluation of the safety of the postbiotic preparation for the target host regarding its intended use.

Postbiotics offer several distinct benefits over probiotics [28]. Their non-living status leads to improved shelf stability and resilience across a wider spectrum of pH and temperature conditions and eliminates concerns regarding antibiotic resistance or the form conditions and amines. The specific chemical structures allow for standardized production and may enhance their antimicrobial capabilities, rendering them a promising substitute or complement to live probiotics. Moreover, they can be utilized alongside packaging materials designed to lessen oxidative stress, which in turn enhances the quality of the product [29]. The composition of postbiotics yields positive effects for both the food product and consumer [28]. Furthermore, the suppression of ROS and the decrease in OS have been demonstrated, suggesting a considerable antioxidant potential [6,30,31].

Several genera of LAB have been identified as significant sources of postbiotics [32]. Information about several of these, including *Lactobacillus*, *Lactococcus*, *Lactiplantibacillus*, *Ligilactobacillus*, *Levilactobacillus*, *Limosilactobacillus*, and *Latilactobacillus*, has been published [29,33,34,35,36].

The beneficial properties of cell-free supernatants (CFSs) derived from LAB have been demonstrated [24,37,38,39]. Similar activity has been observed for exopolysaccharides [34,40,41,42], cell wall fragments (CWF) [43,44], antioxidant enzymes [36,45,46], phenolic compounds [29], and organic acids and short-chain fatty acids [32].

## 3. Lactic Acid Bacteria and Their Relationship with Oxygen

Although LAB are generally considered anaerobic microorganisms, many species can also practice aerobic respiration, which involves the use of oxygen and the requirement of hemin and menaquinone. The ability to use oxygen promotes their growth, survival, and industrial usefulness, allowing them to prosper in a range of environments and undertake more complex metabolic activities than simple fermentation [47].

LAB respiratory metabolism is heavily reliant on the environment, as these bacteria require exogenous heme and menaquinone. Many of the LAB species lack enzymes necessary for heme production, and some of them do not possess the *menFDXBEC* gene, which is responsible for menaquinone biosynthesis [48]. In addition, they express cytochrome oxidase (so-called quinol oxidase CydAB), which functions effectively at low oxygen concentrations, removing the oxygen from the surrounding environment [49]. Unlike typical aerobic bacteria, LAB demand a sugar-based carbon source along with glycolytic activity for the recycling of NADH by NADH oxidase generated in the respiratory chain.

**Figure 1:**
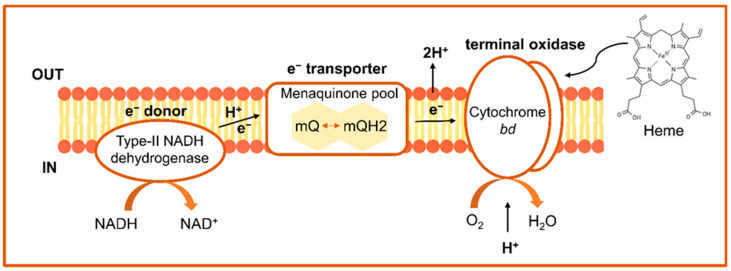
Key elements and processes of respiratory metabolism in lactic acid bacteria.

Several strains belonging to genera such as *Enterococcus*, *Lactococcus*, *Leuconostoc*, and *Weissella* have the ability to produce the (ubi/mena)quinone complex (encoded by *menFDHBEC*, *menA* and *menG*/*UbiE* genes) and generate a respiratory chain in the presence of hemin alone [47,48,50]. This chain contains NADH dehydrogenase that functions as an electron donor; menaquinone, which operates as a quinone shuttle; and haem-binding cytochrome bd-I oxidase, which serves as a terminal oxidase (Figure 1) [51].

According to Lechardeur et al. [48], some LAB encode different genes involved in the heme biosynthetic pathway. However, no single strain has been found to encode the entire gene complex. For example, *Lb. reuteri* possesses early genes involved in the synthesis of vitamin B12, while *L. lactis* encodes late genes that facilitate the incorporation of iron into protoporphyrin. Furthermore, many LAB can utilize menaquinone (i.e., Vit. K_2_) from the surrounding environment because they lack genes encoding menaquinone synthesis [52,53]. Duwat et al. [50] noted that *L. lactis* and *Enterococcus faecalis* synthesize the enzyme ferrochelatase (HemH), which catalyzes the addition of iron to protoporphyrin-IX (PPIX), resulting in the formation of heme.

Published data based on the highly conserved *recA* gene indicate that aerobic respiration does not exhibit species-dependent distribution (Table 1) [48]. Huycke et al. [54] reported that *E. faecalis* possesses genes necessary for respiration, whereas the closely related species *E. faecium* lacks these genes.

## 4. Oxidative Stress and Cell Response in Probiotic LAB

### 4.1. ROS and Oxidative Stress Generation

Despite the tolerance of LAB to oxygen, its presence in the environment is associated with the formation of ROS. The enzymes NADH oxidase (Nox), pyruvate oxidase (Pox), and lactate oxidase (Lox), located in LAB cells, caused the breakdown of oxygen [60,61]. This process generates ^•^O_2_^−^, OH^•^, ^1^O_2_, and ozone (O_3_) [62]. Of these, ^•^O_2_^−^ and OH^•^ are free radicals containing unpaired electrons. ROS that lack unpaired electrons, such as H_2_O_2_ and O_3_, are not classified as free radicals, although they are strong oxidizers.

The monovalent reduction of O_2_ leads to the formation of ^•^O_2_^−^, followed by the reduction of a second electron to generate H_2_O_2_. Through the Haber–Weiss reaction, ^•^O_2_^−^ reacts with H_2_O_2_ to form HO^•^ [63]. The superoxide radical has an extremely short half-life (10^−5^–10^−6^ s). Its role is connected to (i) the site of production in the cell, (ii) the capacity for protonation, and (iii) interaction with a compatible substrate [64]. In microaerophilic bacteria such as LAB, an intracellular source of H_2_O_2_ is the superoxide radical dismutation reaction, or the two-electron reduction of oxygen in reactions catalyzed by Nox, Pox, and Lox [60]. H_2_O_2_ is an unstable molecule with a half-life of 10^9^ s that accumulates in relatively high concentrations in cells due to its extremely slow rate of reaction with biomolecules. The hydroxyl radical is one of the most reactive radicals present in biological systems (half-life 10^−8^ to 10^−9^ s), exerting a direct effect near its point of origin, damaging the first molecule it encounters [65]. Singlet oxygen is a non-radical variant of oxygen, referring to the oxygen molecules where one of the two free electrons is in a higher energy state, leading to the production of free radicals through the energy transfer.

In *Lb. acidophilus* and *Bifidobacterium* spp., two types of NADH oxidases have been reported: NADH-H_2_O_2_ oxidase and NADH-H_2_O oxidase, which catalyze the reduction of O_2_ to H_2_O_2_ (R1) and H_2_O (R2), respectively. Furthermore, NADH oxidase has the potential to cause incomplete reduction of oxygen, producing ^•^O_2_^−^ that can dismutate to H_2_O_2_ or OH^•^ (R3) [66,67].

(R1) $$NADH+H^{+}+O_{2}\overset{NADH-H_{2}O_{2}\;oxidase}{\to }{NAD}^{+}+H_{2}O_{2}\;\;\;\;\;\;$$

(R2) $$2\;NADH+{2H}^{+}+O_{2}\overset{NADH-H_{2}O\;oxidase}{\to }{NAD}^{+}+{2\;H}_{2}O\;\;\;$$

(R3) $$NADH+2\;O_{2}\overset{NADH\;oxidase}{\to }{NAD}^{+}+H^{+}+2\;O_{2}^{-}\;\;\;\;\;\;\;\;\;\;$$

The overproduction of H_2_O_2_ has been observed in *Lb. delbrueckii* subsp. *bulgaricus* and *Lb. plantarum* ATCC 8014^T^ as a result of O_2_ reduction by a NADH-dependent oxidase [68,69]. Stolz et al. [70] proposed that the accumulation of H_2_O_2_ under aerobic conditions and in the absence of catalase results in the activation of NADH-dependent peroxidases, relying on persistent metabolism to produce the needed NADH. Intracellular H_2_O_2_ has been shown to react with Fe^2+^, thereby generating the highly reactive hydroxyl radical (HO^•^) through the Fenton (R4) and Haber–Weiss (R5) reactions. The first radical in the chain, ^•^O_2_^−^, affects cell proteins containing iron–sulfur clusters and enhances the Fe^2+^ level, stimulating the Fenton reaction [60].

Fe3++O2−•↔Fe2++O2−

(R4) $${Fe}^{2+}+H_{2}O_{2}\to {Fe}^{3+}+{HO}^{•}+{OH}^{-}\;\;Fenton\;reaction$$

(R5) $$O_{2}^{-}+H_{2}O_{2}↔O_{2}+{HO}^{•}+{OH}^{-}\;\;\;\;Haber-Weiss\;reaction$$

Under conditions of increased oxygen concentration, the process of ROS formation is accelerated, resulting in the manifestation of OS in cells, including probiotic LAB. The factors that cause stress are extremely diverse. Among them, the availability of oxygen, temperature, osmotic pressure, pH levels, ethanol concentration, etc., originates from environmental sources. The presence of bile, antimicrobials, acidity, starvation, or low nutrient availability can also be significant stressors for probiotic LAB [71]. What these factors have in common is the generation of elevated levels of ROS. It has been reported that increased levels of dissolved oxygen during the cultivation of several strains of *Lb. acidophilus* and *Bifidobacterium* spp. caused OS [54]. Endogenous ROS generation has been evidenced as a key reason for OS realization in *Lb. johnsonii*/*gasseri* strains.

**Figure 2:**
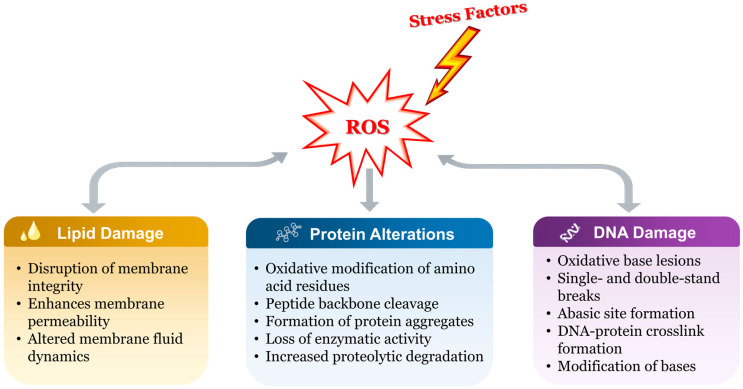
Oxidative damage to intracellular molecules.

The fundamental cause of OS realization is the balance between the generation and elimination of ROS. The overproduction of ROS can disrupt this balance in favor of oxidants in cells, resulting in damage to biomolecules, including lipids, proteins, and nucleic acids (Figure 2) [64]. Probiotics passing through the intestinal tract are subjected to high levels of ROS, which have the potential to harm all cellular components [72]. Despite the extant data concerning the markedly enhanced growth of *L. lactis* under aerobic respiration, a number of studies have been published on the negative consequences of oxygen in lactic acid bacteria [73].

Although the DNA molecule is generally stable, it is susceptible to interactions with reactive oxygen species, which can cause various types of damage. ROS can remove electrons from the DNA chain and transform it into an unstable molecule. Effects such as the breakage of one or both strands, the loss or modification of nucleotides resulting in subsequent mutations, and the covalent binding of a protein molecule have been proven to occur [64,74]. HO^•^ is capable of causing DNA fragmentation by breaking the phosphodiester bonds in the molecule. The effect of ^•^O_2_^−^ on DNA damage has been reported for *Lc. lactis* grown under aerobic conditions [75].

Many forms of ROS can harm intracellular proteins in LAB by various mechanisms. The most common reactions include the removal of hydrogen, transfer of electrons, fragmentation, rearrangement, disproportionation, and substitution in amino acids, peptides, and proteins, among others. HO^•^ has the capacity to damage proteins, which can lead to a reduction in ATP and, as a result, a decrease in energy levels within the LAB cells [60,76]. It has been reported that H_2_O_2_ directly damaged enzyme proteins containing cysteinyl residues, inactivating their activity [77]. Similar results have been reported for *Lb. johnsonii* NCC 533 strain [78].

In addition, other molecules in LAB, such as lipids, are also susceptible to oxygen [60]. It has been established that lipid oxidation of membranes is the primary deleterious effect of ROS attack on lipids [79]. Many serious damages have been found in cellular lipids within the plasma membrane of LAB [60]. Unsaturated linoleic and linolenic fatty acids have been identified as the most sensitive to oxidation in LAB [80].

Lactic acid bacteria experience significant metabolic disruptions when exposed to stress conditions. For instance, LAB can modify specific alternative sources of pyruvate, consume different carbon sources, engage the proteolytic system, and enhance the catabolism of free amino acids. According to Zhai et al. [81], the rise in oxidative stress modifies the carbon source profile of *Lb. plantarum* and stimulates glycolysis, thereby producing more ATP.

### 4.2. Cell Response to Oxidative Stress

**Figure 3:**
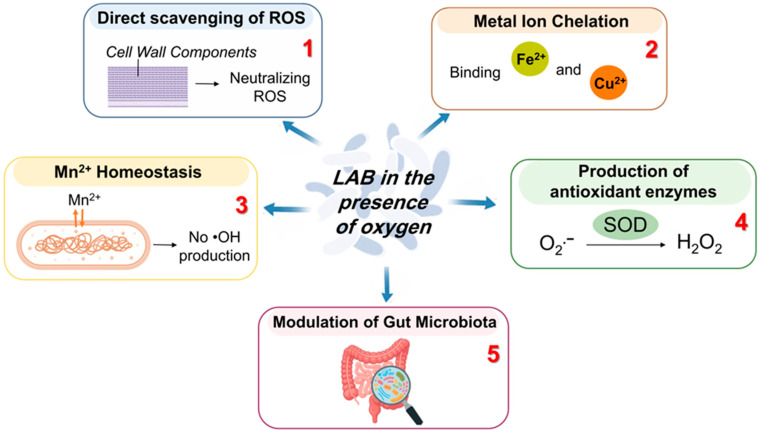
Cell response mechanisms to oxidative stress.

To overcome the deleterious effects of ROS, LAB have developed various responses to OS, depending on the levels and types of antioxidative mechanisms available. As facultative aerobes, they possess an incomplete antioxidant system, including the production of antioxidant compounds and the activation of redox regulatory and repair systems [60,82]. Published reports demonstrated that the aerobic cultivation of some LAB in the presence of haem and menaquinone resulted in increased biomass content due to the formation of a respiratory electron transport chain [56,83]. There are several closely related mechanisms allowing them to neutralize ROS (Figure 3).

- Direct counteraction of ROS (Figure 3, Point 1). LAB can directly scavenge some ROS, such as ^•^O_2_^−^, H_2_O_2_, HO^•^, using several mechanisms. They can prevent the generation of ROS, break the chain of their generation, quench them, or transform H_2_O_2_ to H_2_O through the dismutation of ^•^O_2_^−^. For this purpose, LAB utilize cell wall components such as peptidoglycans, exopolysaccharides, teichoic and lipoteichoic acids, as well as protein.
- Chelation of metal ions (Figure 3, Point 2). Certain structures within the cell walls of LAB facilitate the chelation of metal ions (e.g., Fe^3+^ and Cu^2+^) via the Fenton reaction, thereby decreasing their availability and the opportunity for them to initiate a chain of radical generation [65].
- Mn^2+^ homeostasis and superoxide tolerance (Figure 3, Point 3). Many *Lb. casei* and *Lb. plantarum* require the accumulation of Mn^2+^. Despite functioning as a cofactor for several enzymes, these ions protect cells from the harmful effects of ROS [84]. Intracellular manganese participates in the scavenging of ^•^O_2_^−^ under aerobic conditions.
- Production of antioxidant enzymes (Figure 3, Point 4). LAB can produce enzymes with antioxidant activity, such as superoxide dismutase, haem-dependent and haem-independent catalase, NADH oxidase, NADH peroxidase, and glutathione reductase [56,85].
- Modifications on the metabolite level (Figure 3, Point 5). Under aerobic conditions, a significant number of probiotic LAB have been observed to reduce the production of lactic acid while simultaneously increasing the synthesis of NADH oxidase and NADH peroxidase [86]. For example, *Lb. plantarum* required NADH dehydrogenase for the oxygen respiration [47]. In the presence of oxygen, *L. lactis* exhibited an inhibitory effect on lactic acid fermentation, utilizing cytochrome *bd* oxidase-mediated NADH oxidation within the electron transport chain [87]. Consequently, an alteration in glycolytic flux towards the synthesis of acetate, ethanol, acetoin, diacetyl, and CO_2_ has been observed [85].

## 5. Antioxidants and Antioxidant Defense in LAB

**Figure 4:**
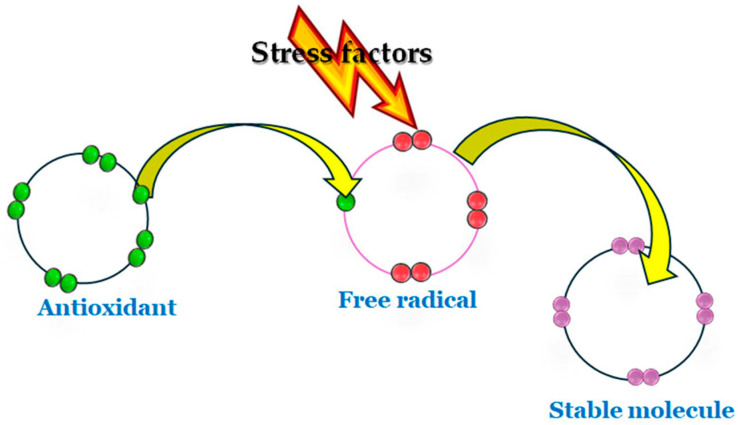
Antioxidant action against free radicals.

Antioxidants are described as “any substance that delays, prevents, or removes oxidative damage to a target molecule” [88]. They are stable molecules that can donate or accept an electron to each free radical, thereby restoring their paired state (Figure 4).

**Figure 5:**
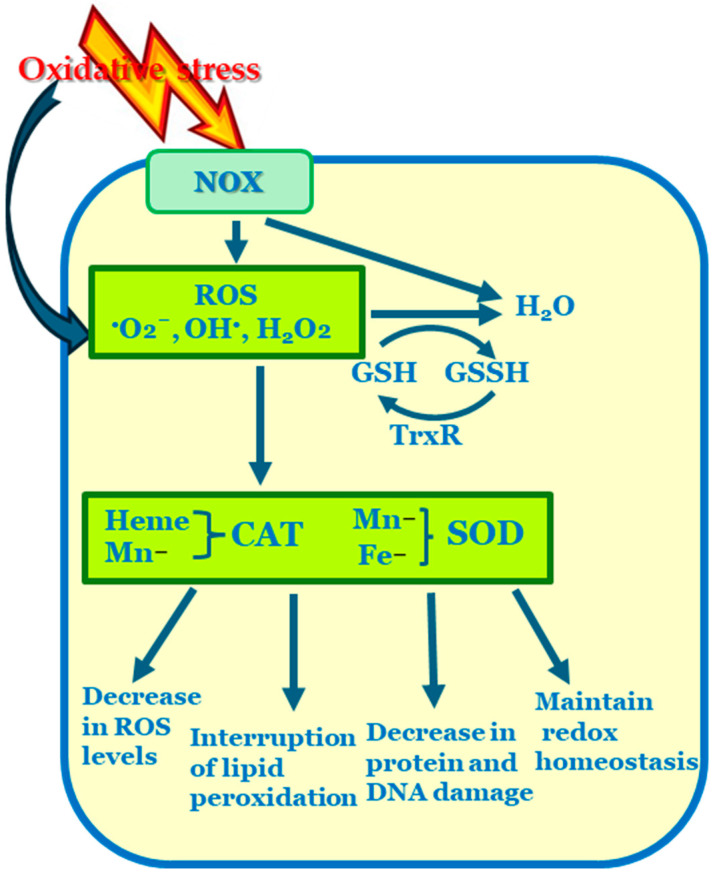
Antioxidant systems in LAB strains.

The effectiveness of the cellular defense system’s antioxidant activity depends on the strategy employed, including prevention, scavenging and destroying radicals, repairing damage, and adaptation (Figure 5). Antioxidant activity involves decreasing the ability of ROS to inflict injury or interacting with ROS to prevent the cascade of their production before essential cellular components are damaged. Furthermore, antioxidants can be categorized into natural and synthetic types, enzymatic and non-enzymatic forms, low- and high-molecular-weight structures, intracellular and extracellular locations, and water-soluble and lipid-soluble varieties [89].

### 5.1. Enzymatic Antioxidant Defense in Probiotic LAB

**O_2_-consuming enzymes.** It has been demonstrated that some probiotic LAB possess O_2_-consuming enzymes participating in the cell response against OS. These enzymes, including NADH oxidase (NOX), pyruvate oxidase (POX), and lactate oxidase (LOX), facilitate the rapid elimination of oxygen and are crucial for maintaining the intracellular redox balance [60]. NOX plays a key role in aerobic growth. There are two forms of NOX: NADH-H_2_O_2_ oxidase (NOX-1) and NADH-H_2_O oxidase (NOX-2). These can be found in LAB strains, either separately or together. NOX-2 enables a direct four-electron reduction to H_2_O, bypassing the two-electron reduction of O_2_ to H_2_O_2_ [82].

POX and LOX also contributed to the accumulation of H_2_O_2_ in several LAB strains growing in the presence of O_2_ [78,90,91]. Both enzymes are expressed throughout growth; however, POX is responsible for producing most of the H_2_O_2_ in the early and logarithmic phases, whereas LOX predominantly contributes to H_2_O_2_ production in the stationary phase. In addition, POX is highly effective in catalyzing the reduction of H_2_O_2_ to H_2_O in *Lb. casei* under oxidative stress conditions [90].

$$NADH\;peroxidase:\;\;NADH+H^{+}+H_{2}O_{2}\to {2HO}_{2}+{NAD}^{+}$$

A lack of NADH oxidase production decreases the growth and oxidative stress tolerance of the *Fructilactobacillus sanfranciscensis* mutant [92]. This enzyme has been reported to play a beneficial role in several species of bifidobacteria, such as *Bifidobacterium longum* [93] and *B. animalis* [94]. Concurrently, LAB can express the NADH-oxidase/peroxidase system, which facilitates the metabolism of molecular oxygen and enhances ATP production. Similar results have been published for *Lb. panis* PM1 [95], *B. longum*, and *B. animalis* subsp. *lactis* [96], among others. Reduction of O_2_ to H_2_O_2_ with NADH oxidase has been demonstrated in *Lb. delbrueckii* and *Lb. plantarum* [68,97]. Shimamura et al. [98] also demonstrated the protective role of NADH oxidase and NADH peroxidase in *B. infantis*, *B. breve*, *B. adolescentis*, and *B. longum* against oxygen toxicity.

**Superoxide dismutase** (EC 1.15.1.1, SOD) is the main enzyme in the first line of antioxidant defense. This enzyme catalyzes the dismutation of ^•^O_2_^−^ to molecular oxygen and H_2_O_2_ according to the following equation [85,99].

O2−•+O2−•+2H+→SODH2O2+O2

SOD is a metalloenzyme and contains a metal atom in the active site, forming four different types of isoenzymes: Fe-SOD, Mn-SOD, Cu/Zn-SOD, and Ni-SOD [90]. Manganese- and Fe-SOD are considered to be evolutionarily more ancient than Cu/Zn-SOD, originating from a common precursor enzyme [100]. During subsequent phases of phylogenetic evolution, these enzymes diverged, leading to the formation of two separate groups based on their amino acid sequences. The evolution of the Cu/Zn SOD family occurred independently, resulting in a notable deviation from the properties of the original two enzymes.

Superoxide dismutase is essential for eliminating oxidative stress in LAB. The basic isoenzymes are Mn- or Fe-containing SODs [101]. However, a cambalistic SOD (Fe/Mn family, containing various amounts of both metals) has also been found in some species [101,102]. According to Feng and Wang [60], MnSOD represents the predominant form found in LAB, in contrast to the relatively infrequent occurrence of FeSOD. The presence of Mn-SOD is determined by the intracellular concentration of Mn^2+^. This isoenzyme has been detected in many members of the genera *Streptococcus* and *Lactococcus* [103]. For example, a high SOD activity has been demonstrated in *Lb. brevis* P68 and *Lb. fermentum* strains E-3 and E-18 [21,104]. The presence of Mn-SOD in *Lb. casei* and *Lb. rhamnosus* strains markedly improved the ROS tolerance of the lactic acid cultures [105,106].

It is noteworthy that Mn-SOD can protect LAB from peroxide stress through the elimination of ^•^O_2_^−^, which disrupts the iron reduction cycle, and decreases the possibility of HO^•^ generation [85]. This suggestion has been confirmed by heterologous expression of the *sodA* gene encoding Mn-SOD of *S. thermophilus* in *Lactobacillus* species that lack this enzyme [107].

**Catalases** (EC 1.11.1.6, CAT) are antioxidant enzymes that are present in nearly all aerobic organisms and a variety of microaerophilic microorganisms [108]. They can break down intracellular H_2_O_2_ before it escapes through the cell membrane in the following reaction:

$${2H}_{2}O_{2}\overset{CAT}{\to }{2H}_{2}O+O_{2}$$

Catalases are categorized into four types based on structure and mechanism: mono-functional haem catalases (classical catalase), catalase-peroxidases (atypical catalase), non-haem catalases (Mn-containing), and minor catalases [109]. In the historical context, LAB were traditionally classified as microorganisms that lacked CAT activity. Nonetheless, there are certain exceptions to this. In the LAB species, heme-dependent or Mn-containing enzymes have been detected. The homotetrameric heme-dependent CAT has been found in cultures grown on the medium supplemented with heme or hematin. Mn-CAT, which does not require heme or hematin, is rarely detected in LAB [85]. The protective effects of Mn-CAT against H_2_O_2_ have been evaluated in *Lb. plantarum* [110], *S. thermophilus* AO54 [111], *Lb. mali* and *P. pentosaceus* [112], and so on. Furthermore, there is evidence to suggest the presence of a heme-dependent catalase in *Lb. pentosus*, *Lb. sake*, *Lb. delbrueckii*, and *Lb. plantarum* WCFS1 [113,114,115]. Engesser et al. [112] characterized CAT in 71 spp. of LAB. The authors detected Mn-CAT activity in *Lb. plantarum*, *Lb. mali*, and *P. pentosaceus*, while heme-CAT has been found in 21 species belonging to the different genera of LAB. It has been demonstrated that the maximum non-heme CAT activity is produced in stationary phase cultures following the complete consumption of glucose.

**Thioredoxin reductase** (EC 1.8.1.9, TrxR) is a flavoprotein with a NADPH cofactor that has the ability to oxidize thioredoxin. The TrxR-selenium complex regulates the entire thioredoxin system and participates in cellular antioxidant defenses [85,89].

The thioredoxin system has been demonstrated to facilitate direct electron transfer to antioxidant enzymes. This process has been shown to enhance their efficiency in scavenging ROS, reduce damaged biomolecules, restore enzyme activity by breaking disulfide bonds, and regulate redox-sensitive transcription factors. The *L. casei* strain Shirota responds to OS by utilizing its thioredoxin (Trx) system to reduce disulfide bonds, thereby preserving the equilibrium of protein dithiol/disulfide bonds [116]. Due to their near-constant exposure to oxidative stress, LAB have evolved genetically encoded antioxidant mechanisms. These mechanisms fundamentally rely on the expression of genes that code for antioxidant enzymes [60,85]. The key genes present in LAB consist of *sodA*, predominantly encoding Mn-SOD; *katA* and *katB*, responsible for encoding catalase; *npr*, *ahpC*, and *ahpF*, which encode peroxidases; *gshR*, which encodes glutathione reductase; and *gor*, which encodes glutathione oxidoreductase [85]. The expression levels of thioredoxin genes depend on conditions related to oxidative stress. As Serata et al. [116] indicated, the *trxB* gene has been demonstrated to play a pivotal role in facilitating the aerobic growth of *L. casei*. Serrano et al. [117] discovered the *trxB1* gene, which encodes *TrxR* in *L. plantarum* WCFS1, and showed that the heterologous overexpression of TrxR significantly improved the strain’s ability to withstand oxidative stress.

### 5.2. Non-Enzymatic Antioxidant Defense in Probiotic LAB

**Glutathione** (γ-Glu-Cys-Gly, GSH) is a non-protein thiol compound that has been found in almost all living organisms. It has the potential to protect cells from ROS and maintain the redox status in the intracellular environment under conditions of abiotic stress [118,119]. Moreover, GSH has also been found to participate in the cell response against oxidative stress of LAB [21,85,89,120]. GSH is essential for the regeneration of other antioxidants, such as vitamins C and E. It preserves –SH groups in their reduced form and contributes to xenobiotic detoxification processes. Adequate intracellular levels of glutathione have been shown to prevent the accumulation of H_2_O_2_, thereby protecting cellular biomolecules from oxidative damage [121]. Comparative proteomic analysis of *L. lactis* SK11 cells, cultivated in the presence or absence of GSH, exhibited enhanced stability of glycolytic enzymes under various stress factors [122]. The authors proposed that glutathione functions as a multifaceted protective agent, promoting LAB survival and cellular integrity.

**Exopolysaccharides** (EPSs) are high-molecular-weight extracellular carbohydrates composed of long-chain biopolymers linked by α- or β-glycosidic bonds [89]. Chemically, EPSs are classified as homopolysaccharides (HoPSs) or heteropolysaccharides (HePSs). They exhibit diverse physiological functions, including antioxidant properties. According to Zhang et al. [123], the majority of LABs are capable of producing EPSs at varying levels, often demonstrating significant antioxidant potential. Similar data have been published for EPS from *S. thermophilus* DSM 24731-EPS_1_, *Lb. delbrueckii* ssp. *bulgaricus* DSM 20081^T^-EPS_5_, *Lb. fermentum* DSM 20049-EPS_6_, *Bifidobacterium longum* ssp. *longum* DSM 200707-EPS_10_ [124].

Strains such as *Lactiplantibacillus* sp. ME2b, *Lactococcus* sp. ME7, *Lacticaseibacillus* sp. ME17 and *Lactobacillus* sp. ME27a, isolated from bovine milk, has been shown to produce EPSs capable of neutralizing free radicals, including DPPH and HO^•^ [125]. Similarly, Yu et al. [126] demonstrated that crude EPSs from *Lb. plantarum* A63 effectively scavenged DPPH, ABTS^+^, and OH^•^ radicals, exhibiting high antioxidant activity.

**Short-chain fatty acids** (SCFAs) are by-products of carbohydrate fermentation. LAB produce acetic acid, propionic acid, and butyric acid, which contribute to the reduction in OS through direct scavenging of ROS [89]. Among them, butyric acid is particularly noteworthy, as it modulates the expression of genes involved in antioxidant enzyme synthesis [127].

**Carotenoids** are natural pigments responsible for red and yellow coloration and are widely recognized for their health-promoting properties, largely attributed to their antioxidant activity [60]. Certain LAB belonging to the genera *Enterococcus* and *Lactiplantibacillus*, known as producers of a deep-yellow C_30_ carotenoid, have been reported to possess resistance to oxidative stress [128]. Analysis of cell-free supernatants from LAB cultures revealed DPPH radical-scavenging activity ranging from 84.1% to 99.5%. Kim et al. [129] found that *Lb. pentosus* strain KCCP11226, harboring the *crtM* and *crtN* genes responsible for C30 carotenoid synthesis, exhibited higher oxidative stress resistance. Under aerobic cultivation, although bacterial growth was slower, carotenoid production increased due to the induced synthesis of mevalonate, a key carotenoid precursor.

Beyond the antioxidant mechanisms described above, additional LAB-derived metabolites—including phenolic compounds, flavonoids, ferulic acid, and histamine—have also been reported to possess free radical-scavenging activity [60]. Furthermore, LABs employ multiple repair mechanisms that facilitate the restoration of oxidatively damaged DNA, proteins, and lipids, thereby collectively enhancing resistance to oxidative stress.

## 6. Antioxidant Potential of Probiotics and Postbiotics

### 6.1. Antioxidant Potential of Probiotic LAB Strains

The antioxidant activity of probiotics and their cell-free fractions (CFS), commonly referred to as postbiotics, has attracted considerable interest in both theoretical research and practical applications. Numerous studies have demonstrated the beneficial effects of probiotics in humans and animals [6,15,130,131]. Probiotic bacteria enhance the host’s cellular response to OS through their intrinsic antioxidant defense systems. These mechanisms include direct scavenging of ROS, metal ion chelation, modulation of cell receptors, and the regulation of signal transduction pathways in eukaryotic cells (Table 2). In addition, probiotics have been observed to stimulate the transcription of antioxidant enzymes [13].

Simultaneously, probiotic bacteria modulate gut microbiota composition by inhibiting the overgrowth of pathogenic microorganisms and influencing intestinal barrier permeability [132]. They have also been reported to contribute to the restoration of the body’s antioxidant status. LeBlanc et al. [45] successfully applied *Lb. casei* BL23, a strain producing SOD and CAT, to alleviate pathological inflammatory processes in mice. Another probiotic strain, *Lb. reuteri*, has demonstrated the ability to colonize the gut and activate specific regulatory systems—such as heat shock protein Lo18, polyphosphate kinase 2, and the regulatory protein RsiR—in response to oxidative stress [133].

**Table 2:** Antioxidant activity of LAB-based probiotic strains.

Probiotic Strain	Source	Reported Antioxidant Functions	Reference
*Lb. casei* BL23		SOD and CAT	[45]
*Lb. plantarum* FC225	Fermented cabbages	SOD and GPX Reduced lipid peroxidation ^•^O_2_^−^, OH^•^, and DPPH radicals scavenge	[23]
*Lb. plantar* MWFLp-182	Feces	SOD, CAT, and GPX	[25]
*Lb. plantarum* MA2	Traditional Chinese fermented foods	SOD, CAT, and GPX	[26]
*Lb. casei* *L. acidophilus*	Microbial collections	Antioxidant peptides	[134]
*Lb. gasseri* ATCC 33323 *Lb. rhamnosus* GG ATCC 53103 *Lb. brevis* ATCC 8287 *Lb. plantarum* ATCC 14917	Microbial collections	Radical scavenging activity Inhibition of peroxyl radical Bacteriocin production	[135]
15 Probiotic isolates	Food and feces	Chelation of Fe ions Decrease of H_2_O_2_ Scavenging of DPPH, hydroxyl radicals, and superoxide anions	[65]
*Lb. plantarum* NJAU-01	Traditionally dry-cured meat product Jinhua ham	Increase the total antioxidant capacity, SOD, GSH-Px, and CAT activity	[27]
*Lb. fermentum*	Culture collection	Scavenging of DPPH, hydroxyl radicals, and superoxide anions	[136]
*Lactobacillus* spp. *S. thermophilus*	Microbial collections	Enhanced SOD activity	[137]
*Lb. acidophilus* (ATCC 4356) *B. longum* (ATCC 15708)	Intestinal tract	Inhibition of linoleic acid peroxidation Scavenge DPPH, hydroxyl radicals, and superoxide anions	[138]
*Lb. fermentum E3*	Fermented goat milk	High glutathione level and MnSOD expression	[21]
*Lb. reuteri* KUB-AC5	Broiler gut	Mn-SOD and Mn-CAT scavenge ROS	[61]
*Lb. rhamnosus*		Mn-CAT scavenging of ROS	[139]
*Lb. rhamnosus*		Mn-SOD scavenging of ROS	[105]
*Lb. alimentarius* 15 M *Lb. sanfranciscensis* 7A *Lb. hilgardii* 51B	Fermented cereal flours	Antioxidant peptides	[140]
LAB isolates LS09, LS10, LS17	Algerian fermented products	Scavenge DPPH, hydroxyl radicals, and superoxide anions	[141]
*Bifidobacterium animalis* 01	Centenarians	Scavenge DPPH, hydroxyl radicals, and superoxide anions	[142]
*Lb. plantarum* ATCC 1443	Microbial collection	Mn-CAT	[110]
*Lb. rhamnosus* *Lb. helveticus*	Traditionally fermented Xinjiang cheese	Scavenge DPPH, hydroxyl radicals	[22]
*P. pentosaceus* RC007	Gastrointestinal content of juvenile rainbow trout	Scavenging of the ABTS^•+^, TEAC, and FRAP radicals	[30]
*Lb. casei* strain Shirota YIT 9029	Microbial collection	Mn ions accumulation	[143]
*Lb. paracasei* ATCC 55544	Microbial collection	Mn ions accumulation	[144]
*Lb. plantarum* WCFS1.	Microbial collections	Mn ions accumulation	[115]
*Lb. ramnosus*	Microbial collections	Mn ions accumulation	[145]

Probiotic LABs are capable of scavenging defined amounts of ROS and protecting against free-radical-induced damage, thereby mitigating oxidative stress. The antioxidant activity of *Lb. plantarum* FC225 has been attributed to its capacity to neutralize ^•^O_2_^−^, OH^•^, and DPPH radicals. LAB strains with probiotic properties, isolated from fermented foods and feces, have demonstrated strong antioxidant capacity by scavenging DPPH^+^, ^•^O_2_^−^, and OH^•^ radicals, as well as chelating ferrous ions [65]. Comparable findings have been reported for *Lb. fermentum* [136], three LAB isolates (LS09, LS10, and LS17) from Algerian fermented products [141], *B. animalis* 01 isolated from centenarians [142], and intestinal lactic acid bacteria *B. longum* (ATCC 15708) and *Lb. acidophilus* (ATCC 4356) [138].

Similarly, *Lb. plantarum* FC225 isolated from fermented cabbages exhibited inhibitory activity against ^•^O_2_^−^, OH^•^, and DPPH^+^ radicals [23]. Four LAB species (*Lb. gasseri* ATCC 33323, *Lb. rhamnosus* GG ATCC 53103, *Lb. brevis* ATCC 8287, and *Lb. plantarum* ATCC 14917) have been shown to scavenge ROS and prevent peroxyl radical-induced DNA damage [135]. Furthermore, the production of bacteriocins may contribute to their overall antioxidant capacity. *Lb. rhamnosus* and *Lb. helveticus* isolated from traditionally fermented Xinjiang cheese exhibited a high DPPH^+^ and hydroxyl scavenging activities [22]. Rosales Cavaglieri et al. [30] also reported remarkable antioxidant properties of *P. pentosaceus* RC007 isolated from the gastrointestinal contents of juvenile rainbow trout, as evidenced by ABTS^•+^, scavenging activity, and elevated TEAC and FRAP values.

The cell response of probiotic LAB to OS also involves modulation of host enzyme activity, including upregulation of antioxidant enzymes and downregulation of ROS-generating systems. Gao et al. [23] reported that probiotic administration enhanced SOD and GPX synthesis while reducing lipid peroxidation levels in mouse liver homogenates. By increasing the activity of SOD, CAT, and GPX, the probiotic strains *Lb. plantarum* C88 and MA2 [25,26] or *Lb. brevis* MG000874 [146] enhanced the health of elderly mice exposed to OS. Elevated SOD activity and increased cellular content of GSH and GSSG have also been reported for *Lactococcus* strains [137].

The probiotic strain *Lb. fermentum* E-3 exhibited prolonged survival associated with high glutathione content and Mn-SOD expression [21]. Similarly, Watthanasakphuban et al. [61] indicated that Mn-SOD and Mn-CAT expression play a key role in mitigating ROS effects in probiotic *Lb. reuteri* KUB-AC5. Several LAB species, such as *Lb. plantarum*, *Lb. sakei*, and *Lb. plantarum* ATCC 1443, possesses manganese-dependent antioxidant enzymes such as Mn-pseudocatalase and Mn-SOD [110,139]. In vivo studies have confirmed high SOD activity in animals administered *Lb. brevis*, *Lb. acidophilus*, and *B. animalis* subsp. *lactis* [147].

The antioxidant capacity is strongly associated with intracellular manganese accumulation [84,89]. While Fe^3+^ and Cu^2+^ promote hydroxyl radicals *via* the Fenton reaction, Mn^2+^ facilitates superoxide anion elimination during aerobic growth [148]. Manganese ions are involved in numerous cellular processes, necessitating tight regulation of their intracellular concentration. This regulation is achieved through the coordinated activity of metal transporters, transcription factors, and oxidative stress-responsive riboswitches. Mn-SOD requires manganese as an essential cofactor [99]. Interestingly, LAB lacking classical SOD enzymes often accumulate high intracellular Mn^2+^ levels, which functionally substitute for SOD activity. The ability to accumulate manganese is frequently associated with the absence of genuine superoxide dismutase activity [143]. Non-enzymatic manganese-mediated oxidative stress management is common in LAB species that lack SODs and/or catalases. For example, *Lb. paracasei* ATCC 55544 retains high viability during storage due to its capacity to accumulate manganese ions acting as free radical scavengers [144]. Enhanced survival under oxidative stress conditions resulting from high intracellular manganese accumulation has also been demonstrated in *Lb. plantarum* [149], *Lb. plantarum* WCFS1 [115], and *Lb. rhamnosus*, *Lb. fermentum* 36, *Lc. mesenteroides* 8293 [145]. The precise mechanism by which Mn^2+^ mitigates oxidative damage in the absence of SOD remains incompletely understood. The prevailing hypothesis suggests that Mn^2+^ forms complexes with low-molecular-weight (LMW) metabolites, which act as ROS scavengers and functionally replace SOD activity [84].

### 6.2. Antioxidant Potential of Postbiotics

Recently, increasing attention has been directed toward the antioxidant properties of postbiotics, including cell-free extracts (CFSs, supernatants), exopolysaccharides released from microbiota, antioxidant enzymes, cell wall components, metabolites, and intracellular extracts (Table 3). Evidence supporting these effects has been obtained from both in vitro and in vivo [15,150].

CFSs derived from LAB are regarded as natural protective agents due to their antimicrobial, antioxidant, and anti-inflammatory properties [37]. They contain residual nutrients and a broad range of bioactive metabolites, including organic acids, bacteriocins, vitamins, extracellular polysaccharides, hydrogen peroxide, enzymes, and ethanol. Moreover, CFSs demonstrated high stability and extended shelf life, which enhances their potential applications in human health and food preservation. Hu et al. [65] reported that CFSs from LAB exhibit significantly greater antioxidant capacity than intact bacterial cells. Similarly, CFS from cultures of *Lb. delbrueckii* ssp. *lactis*, *Lb acidophilus*, and *Lb. casei* displayed higher antioxidant activity than whole-cell preparations [33]. This phenomenon can be attributed to secreted metabolites such as glutathione, butyric acid, folic acid, extracellular polysaccharides, antioxidant peptides, and isoflavone glycosides. Notably, a substantial proportion of antioxidant components in probiotic strains is associated with the cell surface.

Numerous studies have documented the antioxidant properties of LAB-derived supernatant from diverse sources. Huang et al. [37] demonstrated that supernatants and CFSs mixture from *Lb. plantarum* API6, *Lb. plantarum* API1, *Lb. fermentum* 22A, *Lb. salivarius* 26C, *P. acidilactici* 46A, and *Lb. curvatus* L-A1 contain organic acids, volatile compounds, and bacteriocin-like substances. This CFS cocktail exhibited potent antioxidant, antibacterial, and antibiofilm activities. Strong antioxidant capacity has also been reported for CFSs from LAB isolated from endophytic plants (*Lb. plantarum* ngue16, *Lb. plantarum* ng10, *Lb. brevis* w6) [151]. Proton nuclear magnetic resonance (1H-NMR) analysis identified several metabolites contributing to antioxidant activity, including γ-aminobutyric acid (GABA), acetic acid, lactic acid, uracil, uridine, propylene glycol, isopropanol, serine, histidine, and indole-3-acetic acid. Radical-scavenging activity has likewise been demonstrated for CFSs from *Lb. paracasei* B1 and *Lb. plantarum* O24 [39], various *Lactobacillus* species [38], and *Lb. plantarum* O7S1 [24]. Tariq et al. [29] further reported high antioxidant activity of postbiotics (CFSs and cell wall components) from several LAB species, attributing these effects primarily to their phenolic compound content. A comprehensive review of the antioxidant properties of LAB-derived postbiotics has been provided by Aghebati-Maleki et al. [31].

EPS produced by LAB are increasingly recognized as promising antioxidants due to their functional and bioactive properties. As key components of postbiotics, they are considered effective and non-toxic agents with demonstrated antioxidant effects both in vitro and in vivo. Although LAB-derived EPS have been extensively characterized structurally, their specific role as postbiotic effectors remains less thoroughly explored [34]. Multiple studies have demonstrated their ability to inhibit ROS generation [154]. Purified EPSs from *Lb. paracasei* ET-22 exhibited strong postbiotic activity through effective scavenging of ABTS^2+^, DPPH, OH^•^, and ^•^O_2_^−^ radicals [34]. Sulfated EPSs isolated from *Lb. plantarum* WLPL04 significantly inhibited ROS production and lipid peroxidation under H_2_O_2_-induced oxidative stress [35], while also enhancing SOD, CAT, and GPx activities in H_2_O_2_-damaged Caco-2 cells. In vitro studies with EPSs from *W. cibaria* GA44, composed of glucose and rhamnose units, confirmed HO^•^ and ^•^O_2_^−^ scavenging activity [42]. EPSs from *Lb. plantarum* RJF4 demonstrated considerable antioxidant capacity and DPPH radical scavenging activity, along with notable thermal stability (up to 225 °C) [40]. Similar antioxidant properties have been reported for EPSs from *Lb. plantarum* NTMI05 and *Lb. plantarum* NTMI20 [152], *Lb. acidophilus* LA5, and *B. animalis* subsp. *lactis* BB12 [41], and *Lb. plantarum KX041* [155].

The antioxidant activity of postbiotics is largely attributed to antioxidant enzymes such as SOD, GPx, and CAT, which act as the first line of defense for cells [150,156]. High GPx activity has been identified as a key contributor to the antioxidant effects of postbiotics derived from *Lb. plantarum* RG14 [36]. LeBlanc et al. [45] reported that CAT and SOD were produced by engineered *Lb. casei* BL23 effectively reduced the severity of intestinal pathologies. Tomusiak-Plebanek et al. [46] further highlighted the importance of SOD and CAT in the anti-inflammatory and antioxidant effects of *Lb. acidophilus* 900 and *Lb. plantarum* 30B. Additionally, oral administration of postbiotic CAT from *Lactococcus lactis* has been shown to exert a preventative effect against colorectal cancer in mice [153]. Amobonye et al. [32] reviewed the beneficial antioxidant roles of probiotic enzymes, including SOD, CAT, and GPx, produced by *Lc. pseudomesenteroides*, *Lb. paracasei*, *Lb. plantarum*, etc.

Peptides represent another important class of postbiotic components [32]. These biologically active molecules consist of defined amino acid sequences of varying lengths. Specific peptides, such as leucine-leucine-proline (LLP) and valine-tyrosine-proline (VYP), have been identified as antioxidants due to their ability to scavenge free radicals and thereby reduce oxidative stress.

Cell wall fragments (CWFs) further contribute to the functional complexity of postbiotic preparations. Antioxidant activity of CWFs has been demonstrated in postbiotics derived from various LAB species [15]. For example, CWFs from *Lb. casei* CRL-431 have been shown to act synergistically with lipid compounds, with GHS identified as the main contributor to the antioxidant activity of this postbiotic preparation [43]. Additionally, cell wall-bound phenolic compounds isolated from *Lb. plantarum* ATCC 8014 have been reported to enhance the antioxidant capacity of postbiotic formulations [44].

### 6.3. Comparison of Antioxidant Mechanisms of Action

The antioxidant potential of LAB-derived probiotics and their postbiotic functions is strain-specific and context-dependent. The benefits are not uniform across all strains, and individual responses can vary [157]. This observation is supported by Łepecka et al. [89], who analyzed twenty-one LAB probiotic strains belonging to the species *Lb. plantarum*, *Lb. pentosus*, *P. pentosaceus*, and *Lb. paracasei*. Conversely, Abduxukur et al. [158] found that probiotic Lactobacillus strains exhibited the highest hydroxyl and superoxide anion scavenging rate in their IC, followed by CFS.

Furthermore, differences in antioxidant components have been demonstrated between IC, CFS, and metabolites in CFE [159]. The authors reported that the CFS exhibited a higher free radical scavenging rate and greater lipid peroxidation inhibition compared to IC and CFE. Moreover, CFS was distinguished from other fractions by higher antioxidant enzyme activity (SOD and GSH-Px). These strong antioxidant properties and activities can be attributed to metabolites produced during strain cultivation.

According to Wu et al. [160], four LAB probiotic strains (*Lb. plantarum* ZJ316, *Lb. sakei* LZ217, *Lb. paracasei* ZFM54, and *Lb. rhamnosus* ZFM202) displayed varying degrees of H_2_O_2_ tolerance, which correlated with their antioxidant activity. *Lb. plantarum* ZJ316, a strain with strong H_2_O_2_ tolerance, exhibited significant ABTS^+^, DPPH, and HO^•^ scavenging activity. However, its scavenging rate against ^•^O_2_^−^ was weaker, potentially due to less efficient binding of antioxidant components to the superoxide anion target on the intact cell surface. Analysis also revealed significant expression of genes associated with SOD and GSH-Px activity in the CFE. Similar results demonstrating synergistic effects between enzymatic and non-enzymatic systems have been reported for the probiotic strain *Lb. plantarum* GXL94 [161]. The combined utilization of both enzymatic and non-enzymatic antioxidants by LAB has been demonstrated in IC and CFS fractions [162]. In recent years, there has been a notable increase in reports highlighting the ability of lactic acid bacteria to manage oxidative stress through the combined action of these antioxidant systems [89]. For example, Łepecka et al. [89] reviewed such activity for *Lb. fermentum* R6, *Lb. plantarum*, *Lb. paracasei*, *E. faecium*, *Lb. helveticus*, and *W. paramesenteroides.*

## 7. Probiotics and Postbiotics from LAB and Their Impact on Foods

LAB probiotics can be incorporated into a variety of food and beverage products, including both dairy and non-dairy options or administered as supplements. Fermented foods often contain active microbes with genetic similarities to probiotic strains. Research indicates that consuming fermented foods can enhance their functional and nutritional properties by modifying substrates and generating bioactive and bioavailable end-products [72].

**Figure 6:**
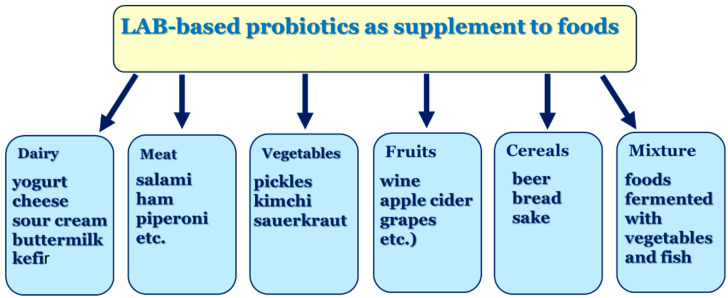
LAB-based probiotics used as supplements to foods.

The application of probiotic bacteria in fermented food production is a significant area of research, particularly regarding their ability to enhance the physical, chemical, sensory, and functional properties of these products, notably their antioxidant qualities [14]. Several probiotic strains with antioxidant properties have been used as supplements to improve the nutritional qualities of foods, thereby mitigating the impact of oxidative stress (Figure 6) [163].

The addition of the probiotic strain *Lb. fermentum* ME-3 to the cheese Pikantne has been shown to increase its total antioxidant properties [164]. Furthermore, novel probiotic cheese supplements exhibiting high radical scavenging activity have been documented, including *Lb. brevis* BJ20 (isolated from traditional Korean fermented food), *Leuconostoc mesenteriodes*, and *Lb. plantarum* (from fermented Chinese cabbage) [165], and *L. lactis* KC24 (from Korean kimchi) [166]. Supplementing cottage cheese with the probiotic strains *Lb. casei* and *Lb. rhamnosus* GG has been demonstrated to enhance its antioxidant and antimicrobial properties [167]. Islam et al. [168] reported four probiotic strains (*Lb. fermentum* SCJ26, *Lb. plantarum* SCJ27, *Lb. fermentum* SCJ28, and *Lb. pentosus* SCJ29 isolated from sugarcane juice as candidates for natural food preservatives.

Probiotics have demonstrated the capability to enhance the quality of fermented milk, with their radical scavenging activity contributing to the antioxidant properties of the products [14]. Consumption of fermented milk containing *Lb. fermentum* ME-3 has been shown to have a beneficial effect on human health, attributed to its substantial antioxidant properties [164]. A comparative analysis demonstrated enhanced total antioxidant activity, plasma GSH, and GPx in yogurt supplemented with probiotic *Lb. acidophilus* LA5 compared to control yogurt [169]. Laali et al. [170] found that incorporating *L. plantarum* in the fermentation of coconut water enhanced the antioxidant properties of the resulting beverage. Similar results have been reported for the supplementation of Cornelian cherry juice fermentation with probiotic *Lb. plantarum* 14917 [171]. Incorporating *Lb. plantarum* CCMA 0743 into the production of functional plant-fermented beverages enhances the antioxidant capacity of the final product [72]. The positive effects of adding LAB to foods to increase their antioxidant capacity have been documented in several types of fermentation. Hunaefi et al. [44] published findings regarding *Lb. plantarum* ATCC 8014 and *Lb. acidophilus* NCFM during red cabbage fermentation. Similar findings have been reported for *Lb. plantarum* in kiwi fruit and cereal grain fermentation [172], as well as for *Lb. plantarum*, *Lb. acidophilus*, *Lb. casei*, *Lb. delbrueckii* subsp. *bulgaricus*, and *Lb. helveticus* in soy product fermentation [132].

Postbiotics have recently garnered increasing interest as nutritional supplements due to their valuable properties, including antioxidant activity. They are potentially more beneficial than probiotics because they can produce similar results even when inactive, thus avoiding the challenges associated with living microorganisms [32]. Unlike live probiotics, postbiotics are unaffected by antibiotics, eliminating the risk of disseminating drug-resistant genes. Furthermore, the bioactive characteristics of postbiotics, including antioxidant activity, and the number of patented postbiotic formulations, promote their application in diverse fields, such as medicine, cosmetics, and the food and pharmaceutical industries. Market investigations project the global postbiotics market to exceed USD 2 billion by 2030, reflecting growing consumer demand for microbiome-supporting products that offer safety, stability, and regulatory transparency [32]. Postbiotics are stable across a wide range of temperatures and pH levels, allowing their addition to foods and ingredients before thermal processing [173]. This offers postbiotic manufacturers technical and financial advantages.

**Figure 7:**
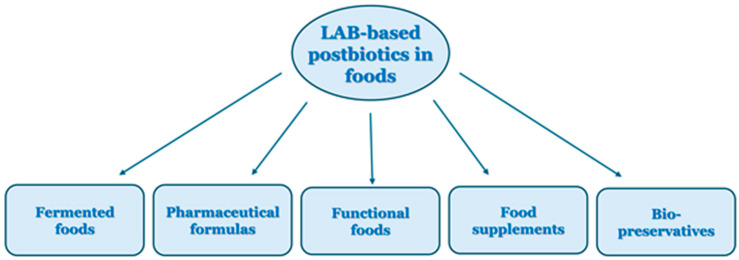
LAB-based postbiotics used in foods.

The integration of postbiotics into diverse food matrices has gained popularity across various categories (Figure 7) [174]. Traditional dairy products, mainly yogurts, cheeses, and fermented milk, have been used as potential carriers. Additionally, postbiotics have been incorporated into a variety of beverages, including fruit juices, functional drinks, and smoothies, providing health benefits without compromising taste or texture. Numerous studies have documented the beneficial effects of postbiotics in food and beverages. They can be added to functional foods and/or drug formulations in controlled amounts under conditions that ensure sufficient survival and effective storage [175]. A postbiotic derived from the novel strain *Lb. plantarum* O7S1 has been recommended as an alternative agent for mitigating oxidative stress in both the food and pharmaceutical industries [24].

The addition of postbiotics derived from *Lb. acidophilus* and *Lb. helveticus* to non-fermented dairy beverages enhanced their antioxidant activity and potential as health-promoting dairy products [176]. Several LAB-based postbiotics have been registered as promoters of antioxidant capacity in various fermented foods. For example, Wu et al. [34] demonstrated that incorporating postbiotics derived from *Lb. plantarum* into fermented blackberry and blueberry juices increased antioxidant activity. Aguilar-Toala et al. [43] characterized the EPS produced by *Lb. rhamnosus* CRL-431 as a natural antioxidant, suggesting it may improve the quality of food products. The authors reported that its addition to milk enhances the physicochemical and sensory qualities of the products. The application of EPS from *Lb. plantarum* YML007 in soybean fermentation extended its shelf life up to two months [177]

Anvar et al. [178] have recommended a combination of plant extract nanoemulsion and postbiotic from *Lb. plantarum* subsp. *plantarum* for butter treatment, which facilitated peroxide radical scavenging and thereby increased its shelf life. Postbiotics derived from a combination of the cell-free extracts and cell wall components of *Lb. plantarum*, *Lb. paraplantarum*, *Lb. brevis*, and *L. lactis* strains have been found to contain high levels of antioxidants and phenolic compounds, as well as acetic and formic acids [29]. These properties contribute to their increased antimicrobial activity against *Escherichia coli*.

*Lactobacillus rhamnosus* and *Lb. reuteri* produce postbiotics with antioxidant qualities that have been described as an efficient natural preservative for use in the meat industry [28]. The results of Jalali et al. [28] confirmed the potential of Lactobacilli-derived postbiotics in red meat preservation. Furthermore, the combination of postbiotics from *Lb. plantarum* and essential oil nanoemulsion from *Thymus daenensis* Celak extended the cold storage of rainbow trout filets due to its antioxidant effects [179]. Ozma et al. [180] reported improved quality of lamb meat slices after treatment with the postbiotic from *Lb. paracasei*.

Postbiotics have emerged as valuable ingredients in functional foods [181,182,183]. These foods are rich in metabolites that have been proven to promote gut health, modulate immune responses, and deliver antioxidant and antimicrobial properties. In addition, they can be employed as a strategy in addressing food allergies [173]. As Wang et al. [184] suggested, the postbiotic of *Lb. plantarum* 70810 containing EPSs has the potential to improve the texture, taste, flavor, and shelf life of functional foods [185]. Similarly, the addition of LAB-derived postbiotics has been shown to significantly improve the antioxidant activity of strawberry juice [186], cheese whey and skim milk during yogurt production [187], bread [188], and sheep’s milk yogurt [189], among other foods. Beyond their antimicrobial effects, postbiotics can inhibit lipid peroxidation, preventing rancidity and discoloration, which are major quality concerns in meat preservation [177].

The strong antioxidant and antimicrobial properties of LAB postbiotics have led to their use as natural preservatives against foodborne pathogens [190]. The application of these natural compounds for bio-preservation is gaining attention as a viable alternative to chemical preservatives. Their compatibility with modern processing technologies, combined with their natural origin, makes them exceptionally well-suited for inclusion in clean-label preservation strategies [191]. The presence of enzymes and non-enzyme antioxidants in postbiotics plays a pivotal role in scavenging ROS, slowing lipid oxidation and protein damage, and mitigating OS in foods [174]. Evidence supports their efficacy in various food sectors, including dairy, meat, beverages, and plant-based foods [192]. Avchi et al. [193] proposed that postbiotics function as lipid peroxidation inhibitors, helping to prevent rancidity and discoloration in meat products. Furthermore, the CFS of three probiotic LAB strains (*Lb. sakei*, *Lb. plantarum* and *P. pentosaceus*), employed as starters in ] sausage fermentation, exhibited a significant rate of radical scavenging and inhibition of lipid peroxidation, thereby improving the quality of the meat products [194]. Comparable results regarding the antioxidant properties of CFS from *Lb. plantarum* and *L. lactis* subsp. *lactis*, used as starter cultures in fermented meat products, have been obtained Bilecen Şen [195].

Reported data indicate that the antioxidant potential of postbiotics can also be exploited for incorporation into oral supplements for humans and animals [6,173,196]. Diets containing LAB-based postbiotics can improve the antioxidant status of sows subjected to heat stress [197], post-weaned lambs [36], and young calves [198].

## 8. Conclusions and Future Scientific Challenges

A thorough analysis of over 180 scientific publications underscores the strategic importance of lactic acid bacteria (LAB) as microorganisms with well-established probiotic and postbiotic potential, including significant antioxidant activity. Recent research highlights the specific adaptations of LAB to utilize oxygen respiration and the mechanisms they employ to mitigate oxidative stress by managing generated free radicals. LAB have developed specialized stress response mechanisms involving both enzymatic and non-enzymatic antioxidant components. These include direct scavenging of reactive oxygen species (ROS), the production of antioxidant enzymes, the synthesis of bioactive metabolites, and the modulation of cellular redox status. Moreover, the incorporation of LAB-derived probiotics and postbiotics into food matrices contributes to improved oxidative stability, extended shelf life, and enhanced functional value.

Despite considerable progress in this field, several key scientific challenges persist. A primary obstacle is the lack of standardized methodologies for evaluating antioxidant activity across in vitro, in vivo, and cell-based models, which hinders comparability between studies. International harmonization of protocols for assessing the antioxidant capacity of probiotics and postbiotics is therefore urgently needed. Furthermore, much of the current evidence is derived from laboratory models. Well-designed, controlled clinical trials demonstrating antioxidant effects in humans remain limited, particularly concerning dose–response relationships, bioavailability, and long-term safety.

While key antioxidant enzymes and metabolites have been identified, the detailed molecular mechanisms regulating redox homeostasis in LAB are only partially understood. Integrating genomics, transcriptomics, proteomics, and metabolomics approaches is crucial to achieve a deeper understanding of cellular responses to oxidative stress. The effects of LAB and their postbiotic metabolites are also known to vary depending on the food matrix (e.g., dairy, plant-based, or meat products). A more comprehensive understanding of the interactions between bacterial metabolites and food components (lipids, proteins, polyphenols) is required, as these interactions may either enhance or diminish antioxidant efficacy. Finally, individual differences in gut microbiota composition may influence the effectiveness of probiotics and postbiotics. Future research should therefore explore the relationship between the antioxidant potential of LAB and personalized nutrigenomic strategies.

## Figures and Tables

**Table 1:** Aerobic respiration among LAB required exogenous heme, or heme and menaquinone.

LAB Required Exogenous Heme	Reference	LAB Required Exogenous Heme and Menaquinone	References
*E. faecalis* *E. casseliflavus* *E. gallinarum* *E. italicus* *Eremococcus coleocola* *L. lactis* *L. garviae* *Lc. argentinum* *Lc. citreum* *Lc. fallax* *Lc. gasicomitatum* *Lb. kimchii* *Lc. mesenteroides* *Weissella cibaria* *W. paramesenteroides* *Streptococcus agalactiae* *S. dysgalactiae* *S. parauberis* *S. pseudoporcinus* *S. uberis*	[47]	*Oenococcus oeni* *S. agalactiae* *S. dysgalactiae* *S. parauberis* *S. pseudoporcinus* *S. uberis* *Lb. santri* *L. brevis* *L. buchneri* *Lb. coryniformis* *Lb. crispatus* *Lb. gasseri* *Lb. fermentum* *Lb. gasseri* *Lb. johnsonii* *Lb. oris* *Lb. paracasei* *Lb. plantarum* *Lb. reuteri* *Lb. rhamnosus* *Lb. ultunensis* *Lb. vaginalis*	[47]
*Lc. mesenteroides* *O. oeni* *L. lactis*	[48]		
		*Lb. johnsonii* *Lb. reuteri* *L. fermentum* *L. plantarum* *L. brevis*	[48]
*L. lactis MG1363*	[50]		
*W. minor* *Lc. citreum* *L. pseudomesenteroides*	[55]		
*L. mesenteroides* subsp. *cremoris*	[56]	*S. agalactiae*	[48,52]
*S. entericus* *Lb. paralimentarius* *S. entericus*	[57]	*L. casei*	[47,58]
*Lc. gasicomitatum*	[59]	*Lb. salivarius*	[47,48]
		*Lc. garvieae* *Lb. plantarum*	[59]

**Table 3:** Overview of antioxidant activity of postbiotics derived from LAB probiotic strains.

LAB Strain	Source	Postbiotic Component(s)	Reported Antioxidant Functions	Reference
*Pediococcus pentosaceus*	Culture collection	CFS	ABTS^•+^, TEAC, FRAP scavenge	[30]
*Lb. plantarum* API6	Worker bees Human fecal samples	CFS	DPPH reducing activity	[37]
*Lb. plantarum* API1				
*Lb. fermentum* 22A				
*Lb. salivarius* 26C				
*P. acidilactici* 46A				
*Lb. curvatus* L-A1				
*Lb. plantarum* ngue16	Endophyte plants	CFS	DPPH and FRAP reducing activity	[151]
*Lb. plantarum* ng10				
*Lb. brevis* w6.				
*Lb. paracasei* B1	Polish regional sheep’s milk cheese	CFS	ABTS^•+^, DPPH reducing activity	[39]
*Lb. plantarum* O24				
*Lb. acidophilus*		CFS	DPPH reducing activity	[38]
*Lb. fermentum*				
*Lb. rhamnosus*				
*Lb. casei*				
*Lb. plantarum*				
*Lb. reuteri*				
*Lb. plantarum* O7S1	Fermented olives	CFS	DPPH, hydroxyl, and superoxide radicals reducing activity	[24]
*Lb. plantarum*	Culture collection	CFS (phenolic compounds)	DPPH, ABTS reducing activity	[29]
*Lb. paraplantarum*				
*Lb. brevis*				
*Lactococcus lactis*				
*Lb. delbrueckii* ssp. *lactis*	Stock culture collection	CFS	Radical scavenging activity	[33]
*Lb. acidophilus*				
*Lb. casei*				
*Lb. paracasei* ET-22		EPS	DPPH, ABTS hydroxyls, and superoxide anion reducing activity	[34]
*Lb. plantarum* WLPL04	Woman’s breast milk	Sulfated EPS	Inhibition of the ROS and lipid peroxidation Increase in SOD, CAT, and GPx	[35]
*Weissella cibaria* GA44	Nigerian traditional fermented cassava mash “gary”	EPS	ROS scavenging activity	[42]
*Lb. plantarum* RJF_4_	Rotten jackfruit	EPS	Total AO, DPPH scavenging activity	[40]
*Lb. plantarum* NTMI05	Milk products	EPS	Total AO, DPPH scavenging activity	[152]
*Lb. plantarum* NTMI20				
*Lb. acidophilus* LA5	Culture collection	EPS	Total AO, DPPH scavenging activity	[41]
*B. animalis* subsp. *lactis* BB12				
*Lb. plantarum* KX041	Chinese pickle juice sample	EPS	ABTS, DPPH, hydroxyl, and superoxide scavenging activity	[132]
*Lb. plantarum* RG14,	Culture collection	GPx	Total antioxidant activity, Reduced lipid peroxidation Upregulated antioxidant enzymes	[36]
*Lb. casei* BL23		CAT SOD	Free radical scavenging activity	[45]
*Lb. acidophilus* 900		CAT SOD	Free radical scavenging activity	[46]
*Lb. plantarum* 30B				
*Lc. lactis*	Culture collection	CAT	Free radical scavenging activity	[153]
*Lb. casei* CRL-431	Culture collection	CWF	Total antioxidant activity	[43]
*Lb. plantarum* ATCC 8014	Culture collection	CWF	Total antioxidant activity	[44]

## Data Availability

No new data were created or analyzed in this study. Data sharing is not applicable to this article.

## Abbreviations

The following abbreviations are used in this manuscript:

LAB	Lactic acid bacteria
OS	Oxidative Stress
ROS	Reactive Oxygen Species
^•^O_2_^−^	Superoxide anion
^•^OH	Hydroxyl radical
^1^O_2_	Singlet oxygen
NOX	NADH oxidases
POX	Pyruvate oxidases
LOX	Lactate oxidases,
SOD	Superoxide dismutase
CAT	Catalase
TrxR	Thioredoxin reductase
GPx	Glutathione peroxidase
GSH	Glutathione
GIT	gastrointestinal tract
CFSs	cell-free supernatants
CWF	cell wall fragments
EPS	Exopolysaccharides
SCFA	Short-chain fatty acids
